# Pharmacological Profile of the Sodium Current in Human Stem Cell-Derived Cardiomyocytes Compares to Heterologous Nav1.5+β1 Model

**DOI:** 10.3389/fphar.2019.01374

**Published:** 2019-12-11

**Authors:** Dieter V. Van de Sande, Ivan Kopljar, Ard Teisman, David J. Gallacher, Dirk J. Snyders, Hua Rong Lu, Alain J. Labro

**Affiliations:** ^1^Laboratory of Molecular, Cellular, and Network Excitability, University of Antwerp, Antwerp, Belgium; ^2^Global Safety Pharmacology, Non-Clinical Safety, Janssen R&D, Beerse, Belgium

**Keywords:** arrhythmic, SCN5A, lidocaine, phenytoin, flecainide, quinidine, patch-clamp

## Abstract

The cardiac Nav1.5 mediated sodium current (I_Na_) generates the upstroke of the action potential in atrial and ventricular myocytes. Drugs that modulate this current can therefore be antiarrhythmic or proarrhythmic, which requires preclinical evaluation of their potential drug-induced inhibition or modulation of Nav1.5. Since Nav1.5 assembles with, and is modulated by, the auxiliary β1-subunit, this subunit can also affect the channel’s pharmacological response. To investigate this, the effect of known Nav1.5 inhibitors was compared between COS-7 cells expressing Nav1.5 or Nav1.5+β1 using whole-cell voltage clamp experiments. For the open state class Ia blockers ajmaline and quinidine, and class Ic drug flecainide, the affinity did not differ between both models. For class Ib drugs phenytoin and lidocaine, which are inactivated state blockers, the affinity decreased more than a twofold when β1 was present. Thus, β1 did not influence the affinity for the class Ia and Ic compounds but it did so for the class Ib drugs. Human stem cell-derived cardiomyocytes (hSC-CMs) are a promising translational cell source for *in vitro* models that express a representative repertoire of channels and auxiliary proteins, including β1. Therefore, we subsequently evaluated the same drugs for their response on the I_Na_ in hSC-CMs. Consequently, it was expected and confirmed that the drug response of I_Na_ in hSC-CMs compares best to I_Na_ expressed by Nav1.5+β1.

## Introduction

A major cause of cardiac liabilities is drug-induced arrhythmia by the modulation of different ion channels in the heart. One of the most important targets is the sodium current (I_Na_) that generates the primary depolarizing power determining the upstroke of the action potential (AP) in human atria and ventricular myocytes. Effects on this ion channel should be scrutinized, as inhibition or modulation of it can cause severe adverse drug effects associated with arrhythmia (Lu et al., 2010; Jung and Kwak, 2016). This liability is clearly demonstrated in, e.g., the Cardiac Arrythmia Suppression Trial (CAST) of the late eighties demonstrating the proarrhythmogenic complications associated with I_Na_ inhibitors (Cardiac Arrhythmia Suppression Trial (CAST) Investigators, 1989). Human (induced pluripotent) stem cell-derived cardiomyocytes (hSC-CMs), which express a repertoire of ion channel proteins including their auxiliary subunits, are a promising cell source for human representative *in vitro* models for early cardiac proarrhythmia detection. Although limitations related to maturation of the currently available hSC-CMs may still give some concerns in predicting proarrhythmia, the expression of a repertoire of various ion channel related proteins can clearly be to their advantage over the study of drug effects in heterologous expression systems representing single ion channels.

The cardiac I_Na_ is mainly constituted by coassembly of the voltage-gated sodium Nav1.5 α-subunit, the channel forming protein that is expressed by the SCN5A gene, and auxiliary subunits in a noncovalent manner (Gellens et al., 1992; Isom et al., 1994). It has been demonstrated that these auxiliary subunits can modulate the Nav1.5 channel properties, such as altering the current density and the kinetics of activation and/or inactivation (Abriel, 2010). The auxiliary β1-subunit is well documented to associate noncovalently with the Nav1.5 α-subunit (Zhu et al., 2017), which upon association is known to increase the I_Na_ density by facilitating the integration of the Nav1.5 α-subunit into the cell membrane (Qu et al., 1995). The effects on the kinetics of Nav1.5 are rather minor but they vary between reports, as reviewed by Baroni and Moran (2015). Since the modulating effect of β1 on the pharmacological response of Nav1.5 is less studied (Makielski et al., 1996), expression differences of this subunit in hSC-CMs and heterologous expression models could result in pharmacological differences between both systems. To address this, the effect of β1 on the pharmacological profile of Nav1.5 was first determined in a heterologous expression system and subsequently compared to the pharmacological effect of I_Na_ in Cor.4U hSC-CMs. These hSC-CMs are reported to at least express β1 thus potentially recapitulating the native I_Na_ of ventricular cardiomyocytes (Huo et al., 2017; Moreau et al., 2017).

Drugs that inhibit/modulate Nav1.5 have been classified in three classes (Ia, Ib and Ic) (Harrison, 1986). Whereas the mode of action of these drugs can differ, they are mostly following the modulated receptor hypothesis whereby the channel needs to be in a specific state to be affected by the drug (Hondeghem, 1987). This can lead to a use dependency for some drugs: the more the channel is activated, i.e., the more often it transitions from the closed to the open or inactivated state(s) and back, the stronger is the effect of the drug (Hille, 1977). In this study, five different drugs (selected to represent the three different classes) were evaluated for their effect on the I_Na_ with the aim to i) compare the affinity of I_Na_ inhibitors between hSC-CMs (Cor.4U) and Nav1.5 expressed in heterologous cell lines and ii) determine the contribution of the auxiliary β1 subunit in the pharmacological response of I_Na_.

## Material and Methods

### Cell Culture and Transient Transfection Protocol

COS-7 cells (CV-1 in Origin with SV40 genes) were selected as heterologous expression system because of the absence of reports on the expression of endogenous β1 and Nav channel subunits. Cells were cultured in 20-cm^2^ dishes with 5 ml of DMEM medium supplemented with 4.5 g/l D-Glucose + D-Glutamate, 10% Foetal bovine serum, and 1% penicillin/streptomycin in a Galaxy B incubator (RS Biotech, Irvin, United Kingdom) under 5% CO_2_ atmosphere at 37°C. All products were obtained from Gibco (Thermo Fisher, Waltham, Massachusetts, USA). COS-7 cells were transiently transfected with 5 µg hSCN5A cDNA (cloned in a Pcl 398 vector) per 20 cm^2^ cell culture dishes or cotransfected in a 1:1 ratio with 5 µg hSCN5A and 5 µg hSCN1B (cloned in pRcCMV), using lipo2000 as transfection reagent (Invitrogen, Carlsbad, CA, USA). Transfection was performed on cells of 20-cm^2^ dishes in which the culture reached approximately 70% confluency. 0.5 µg of the eGFP-expressing plasmid was cotransfected to visualize successfully transfected cells under an Eclipse TE2000-U inverted fluorescence microscope (Nikon instruments Europe, Amsterdam, Netherlands) during electrophysiological patch-clamp experiments. After 48-hour incubation of the cells in culture medium with transfection solution at 37°C, the cells were harvested by trypsinization and used for patch-clamp experiments.

hSC-CMs (Cor.4U cells) were purchased from Ncardia (Cologne, Germany) and delivered in liquid nitrogen frozen vials containing 250K (Ax-B-HC02-MPC) or 1M cells (Ax-B-HC02-1M). hSC-CMs were seeded, following the provider’s protocols, on fibronectin coated glass coverslips and stored in a 24-well plate at 37°C and under 5% CO_2_ atmosphere with 1 ml Cor.4U media without antibiotics per well. The fibronectin coating was done by adding 400 µl of a 10-µg/ml fibronectin solution, which was obtained by diluting a 1-mg/ml fibronectin stock in 0.05 M TBS (Tris-buffered saline) at pH 7.5 (Sigma-Aldrich, St.Louis, Missouri, USA) in PBS with Ca^2+^ and Mg^2+^. For accurate voltage-clamp experiments, cells were seeded at low densities to obtain single isolated hSC-CMs on the cover slip. hSC-CMs were stored in an IGO150 cell life incubator (Waltham, Massachusetts, USA) under 5% CO_2_ atmosphere at 37°C. When the hSC-CM cells were frozen at Ncardia, they were at day 31 postdifferentiation. After seeding, the hSC-CMs were cultured for another week before starting voltage-clamp experiments, giving them enough time to recover from thawing. Subsequently, electrophysiological experiments were performed within the following 2 weeks (i.e., day 38 to 52 postdifferentiation).

### Electrophysiology and Applied Pulse Protocols

Whole cell patch-clamp experiments were performed on hSC-CMs and transient transfected COS-7 cells, at room temperature (21°C ± 1°C), using an Axopatch 200B amplifier (Axon CNS Molecular devices, San Jose, CA, USA). Current recordings were, after passing a 5- or 10-kHz low pass filter, sampled at 10 or 20 kHz and digitized using a Digidata 1440A (Axon CNS Molecular devices). Applied voltage protocols and elicited current recordings were controlled and stored using the pClamp10 software (Axon CNS Molecular devices). Patch pipettes were pulled from 1.2-mm quick-fill borosilicate glass capillaries (World Precision Instruments, Sarasota, FL, USA) with a horizontal P-2000 puller (Sutter Instrument Co., Novato, CA, USA) and subsequently heat polished. Pipette resistance varied between 1 and 1.5 MΩ for patching COS-7 cells and between 2 and 4 MΩ for hSC-CMs. The shape of the pipettes for an improved sealing quality on hSC-CM was sharper, resulting in the slightly higher pipette resistance. The glass pipettes were backfilled with an intracellular solution (ICS) containing in mM: KCl 150, NaCl 5, CaCl_2_ 2, MgCl_2_ 5, EGTA 5, HEPES 10, and adjusted to pH 7.2 with KOH. The cells were superfused continuously, at a rate of approximately 1 ml/min, with an extracellular solution (ECS) containing in mM: NaCl 150, KCl 5.4, CaCl_2_ 1.8, MgCl_2_ 1, glucose 15, HEPES 15, Na-pyruvate 1, and adjusted to pH 7.4 with NaOH. These ICS and ECS solutions were used for measuring I_Na_ in both the heterologous COS-7 and hSC-CM cells. Because of its fast kinetics the I_Na_ could be well determined in hSC-CM without the need of selectively blocking other currents. However, to minimize the possible contribution of a small I_to_ K^+^ current, the I_Na_ drug response was determined at −10 mV to have minimal I_to_ activation (Cordeiro et al., 2013). The following drugs were dissolved in a 100% DMSO stock solution: flecainide (40 mM stock, reported pKa of 9.3), quinidine (40 mM stock, reported pKa of 8.56), lidocaine (200 mM stock, reported pKa between 7.6 and 8.0), phenytoin (200 mM stock, reported pKa of 8.33), and ajmaline (200 mM stock, reported pKa of 8.32). Test concentrations were made daily by diluting the stock solutions further and dissolving them in ECS, yielding for each drug concentration tested a final DMSO concentration of 0.1%. Due to solubility, it was not possible to make a phenytoin concentration larger than 300 µM.

Four different voltage protocols were applied. The first (activation) protocol consisted out of 21 sweeps with each sweep composed of four different voltage steps. Starting from a holding potential of −90 mV, a fixed 80 ms conditioning prepulse to −130 mV was applied followed by a 40-ms depolarizing step that varied between +80 and –130 mV. After the depolarizing step, the cell was always clamped back to −90 mV. Intersweep interval was set at 8 s and every new sweep was preceded by a pulse P/-6 subtraction protocol starting from −90 mV holding. This P/-6 protocol allowed subtracting the capacitive currents in the recordings and consequently corrected for leak (**Supplemental Figure 1**).

The second (inactivation) protocol was used to determine the amount of Nav1.5 inactivation and contained 17 sweeps with an intersweep interval of 8 s. After the 100-ms prepulse to −130 mV, the cell was clamped for 500 ms to a voltage between −135 and −55 mV, with 5-mV increments between every sweep. The amount of channel inactivation, induced during the 500-ms step was evaluated during a subsequent 50-ms step to 0 mV, a voltage that opens all channels. Consequently, the amplitude of the current elicited during this 0-mV step was a direct measurement of the fraction of channels that were not inactivated. Thirdly, an inactivation recovery protocol was applied. After inducing channel inactivation with a 500-ms depolarization to −10 mV, the cell was clamped to −130 mV for variable times ranging between 0.5 and 50 ms. Subsequently a short −10-mV depolarization step was applied to determine the number of channels that recovered from inactivation during the brief but time variable −130-mV step. Intersweep interval was 8 s. Finally, a drug wash-in protocol was used (100 sweeps) to monitor the development of current inhibition. After 80 ms at −130 mV, a 40-ms depolarizing step to −10 mV was applied. There was no change in voltage steps between sweeps and sweeps were repetitively applied at 1 Hz. During the intersweep time, a P/-6 protocol was applied and represented currents are the subtracted tracings.

### Data Analysis

Current-voltage relations were obtained by normalizing the peak I_Na_ amplitude to the cell capacitance and plotting these values as a function of the applied potential. Normalized conduction-voltage curves were obtained by approximating the linear part of the current-voltage relation (between +80 and +50 mV) with the function I = Gmax*V to determine maximal conduction Gmax. Dividing subsequently all the data points of the current-voltage relation by the calculated maximum current at that voltage, using the estimated Gmax, yielded the normalized conduction-voltage (GV) relation. Voltage dependence of channel inactivation was obtained by normalizing the currents elicited during the 0-mV test pulse of the inactivation protocol and plotting them as a function of the prepulse potential. The normalized GV relations, which represented the voltage dependence of channel opening and the voltage dependence of inactivation, were fitted with a Boltzmann equation y = 1/(1+exp(-(V-V_1/2_)/*k*)). V_1/2_ is the potential at which 50% of the channels are open or inactivated, and *k* is the slope factor. Time constants of channel inactivation were obtained by approximating the current decay upon activation with a single exponential equation. The drug’s inhibiting effect was quantified by normalizing the steady-state currents upon drug addition to the control values obtained in standard ECS. The remaining current was then plotted against the respective drug concentration resulting in a concentration-effect curve. All drugs were added in the sequential order from the lowest to the highest concentration using a pressurized fast switching perfusion system. Estimate IC_50_ values for lidocaine were obtained by fitting the concentration-effect curves with a Hill function, y = min+((max-min)/(1+(x/IC_50_)^-Hill slope^)). All values are plotted as the mean ± S.E.M., with n the number of cells analyzed. In case of hSC-CMs also the number of independent cell batches is indicated, thus number of cells n/#batch. Recordings were excluded from analysis if the voltage error, originating from series resistance problem, exceeded 5 or 8 mV for the experiments on COS-7 and hSC-CM cells, respectively. One-way ANOVA method for many groups was used to find statistical significance in drug response (amount of current inhibition) between Nav1.5 alone, Nav1.5+β1, and hSC-CMs. P-values lower than 0.01 were considered to indicate statistical significance. Data were analyzed using pClamp10 software (Axon CNS Molecular devices) and the program Sigmaplot 11 (Systat software inc., Chicago, Illinois, USA).

## Results

**Figure 1:**
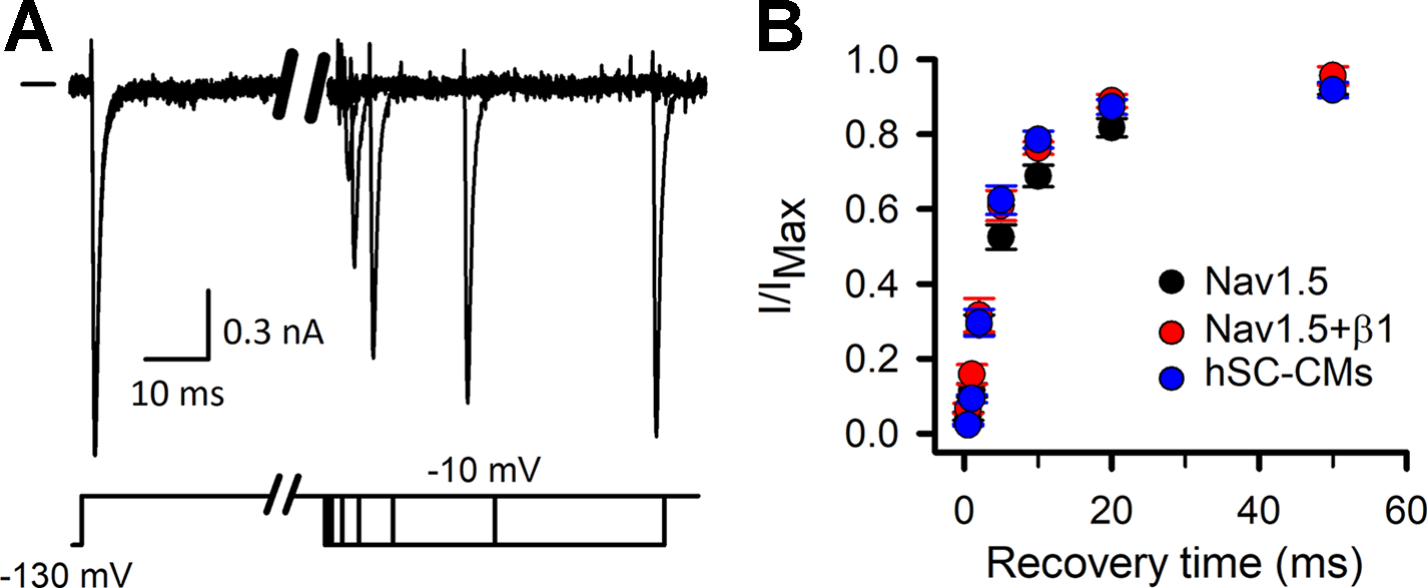
I_Na_ recovery from inactivation: **(A)** I_Na_ recovery from inactivation example traces, chosen from the Nav1.5+β1 model. Currents were elicited with the inactivation recovery pulse protocol as described in material and methods, with horizontal bar at the start indicating the zero current level. Depending on the amount of channels that have been able to recover during the repolarizing step the amplitude of current will increase. **(B)** Fraction of current recovered, obtained by dividing the amplitude of the recovered current to the maximum current level (I/I_MAX_), is plotted as a function of the −130-mV prepulse duration (recovery time). Values are mean ± S.E.M. for Nav1.5 (black, n = 5), Nav1.5+β1 (red, n = 5), and hSC-CM (blue, n/#batches = 5/1).

Before evaluating the response of drugs, the biophysical properties of I_Na_ were characterized in hSC-CMs and compared to I_Na_ of COS-7 cells expressing either Nav1.5 alone (mentioned throughout as Nav1.5 model) or Nav1.5 with its β1-subunit (Nav1.5+β1 model). First, the time-course of recovery from channel inactivation was determined for the three models, as it has been reported that β1 accelerates this recovery process (Zhu et al., 2017). After inducing channel inactivation, the time course of recovery was evaluated at −130 mV (**Figure 1**). Comparing the three models, the Nav1.5 model recovered slightly slower from inactivation than the Nav1.5+β1 and hSC-CM model. Time constants of recovery were 5.90 ± 0.02 ms, 5.06 ± 0.01 ms, and 5.05 ± 0.03 ms for Nav1.5, Na1.5+β1, and hSC-CMs, respectively (n = 5 for each model). After 50 ms repolarization at −130 mV, I_Na_ recovery appeared almost complete in all three models. Therefore, we used for the other pulse protocols a conditioning prepulse to −130 mV longer than 50 ms in duration to recover the channels from inactivation.

**Figure 2:**
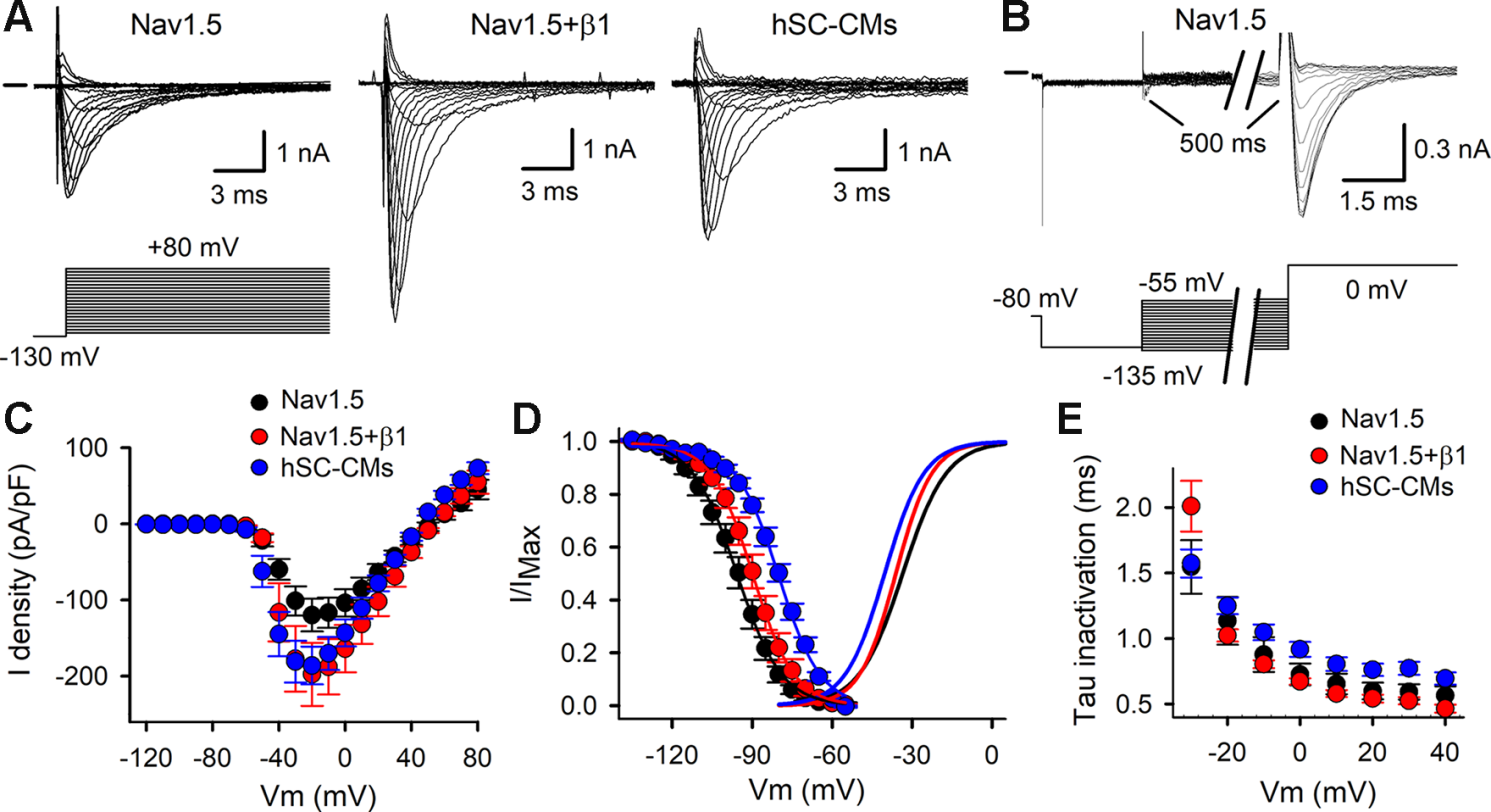
Activation and inactivation of I_Na_: **(A)** Representative I_Na_ recordings elicited with the activation pulse protocol (shown below) for Nav1.5, Nav1.5+β1, and hSC-CM. Zero current level is indicated by horizontal bar at the start. **(B)** I_Na_ recordings to determine the voltage-dependence of channel inactivation elicited with the inactivation pulse protocol (shown below). **(C)** IV curves obtained from analyzing current recordings shown in panel **(A)**. The peak I_Na_ currents were normalized to the cell capacitance and plotted as a function of applied potential. Shown values are mean ± SEM for Nav1.5 (black, n = 20), Nav1.5 + β1 (red, n = 15), and hSC-CM I_Na_ (blue, n/#batch = 17/4). **(D)** Voltage dependence of inactivation obtained from analyzing current recordings shown in panel B. Note, coassembly of Nav1.5 with β1 (red circles, n = 15) shifted the voltage dependence of inactivation toward more depolarized potentials compared to Nav1.5 alone (black circles, n = 17). I_Na_ of hSC-CM (blue circles, n/#batch = 7/2) inactivated at more depolarized potentials compared to Nav1.5 and Nav1.5+β1. The average voltage dependence of activation, obtained from analyzing the IV curves of panel C, are represented with solid lines. **(E)** Plot displays the time constants of inactivation, which were obtained by approximating the current decay from recordings shown in panel A with a single exponential function. No marked differences were observed between Nav1.5 alone (black, n = 12), Nav1.5+β1 (red, n = 14), and I_Na_ in hSC-CMs (blue, n/#batch = 10/2).

Representative I_Na_ recordings of COS-7 cells expressing either Nav1.5 alone or Nav1.5+β1, and from hSC-CMs are shown in **Figures 2A, B**. Comparing the heterologous models, the amplitude of I_Na_ elicited by Nav1.5+β1 was approximately a twofold larger compared to Nav1.5 alone. The current-voltage (IV) plots highlighted the increased current expression of Nav1.5+β1 compared to Nav1.5 (**Figure 2C**), which supports previous findings that β1 facilitates the membrane incorporation of Nav1.5 (Qu et al., 1995; Makielski et al., 1996; Zhu et al., 2017). The current density of Nav1.5 and Nav1.5+β1 at –20 mV amounted to −119 ± 21 pA/pF (n = 19) and −198 ± 41 pA/pF (n = 14), respectively. The normalized conduction-voltage (GV) relation yielded for Nav1.5 a V_1/2_ of −33.5 ± 0.3 mV with a slope factor *k* of 6.9 ± 0.3 (n = 20). For Nav1.5+β1, the V_1/2_ amounted to −36.1 ± 0.6 mV and *k* to 7.3 ± 0.5 (n = 15), indicating that β1 did not alter the voltage dependence of activation significantly (**Figure 2D**). However, the voltage dependence of inactivation was shifted slightly (by +6 mV, p-value <0.001) to more depolarized potentials when β1 was coexpressed, displaying inactivation curves with a V_1/2_ of −95.8 ± 0.3 mV (n = 18) and −89.8 ± 0.2 mV (n = 15) with a slope factor *k* of 8.2 ± 0.3 mV and 6.8 ± 0.5 mV for Nav1.5 and Nav1.5+β1, respectively. Similar to the activation kinetics, no differences were observed in the time constants of channel inactivation between Nav1.5 and Nav1.5+β1 (**Figure 2E**).

After characterizing I_Na_ in both heterologous models, the results were compared to the I_Na_ of hSC-CMs. In hSC-CMs, the peak I_Na_ density was around −30 mV and amounted to -186 ± 25 pA/pF (n/# batch = 15/4). It is important to note that the recordings were done in ECS with normal sodium concentrations (150 mM Na^+^ extracellular). Consequently, I_Na_ amplitudes were in some cells too large to be controlled accurately, because of the accompanying voltage errors, and these recordings were excluded from analysis. Therefore, the reported I_Na_ density is an underestimation. One solution could have been to reduce the extracellular [Na^+^] (**Supplemental Figure 1**), as has been done previously (Lin et al., 2015; Hayano et al., 2017; Moreau et al., 2017). However, because the majority of the data from heterologous expression systems are obtained with normal physiological extracellular [Na^+^], we opted to perform the hSC-CM recordings in similar conditions. After constructing the IV relation of I_Na_ in hSC-CMs, the GV relation was created and displayed a V_1/2_ of −40.2 ± 0.6 mV with a slope factor *k* of 7.4 ± 0.3 (n/#batch = 15/4). Compared to the data from the heterologous models, the voltage dependence of I_Na_ activation was in hSC-CMs shifted slightly toward more hyperpolarized potentials by approximately −5 mV. In contrast, the voltage dependence of I_Na_ inactivation was for hSC-CMs shifted toward more depolarized potentials compared to Nav1.5 or Nav1.5+β1 models (**Figure 2D**), displaying a V_1/2_ of −80.0 ± 0.4 mV (n/#batch = 7/2) with a slope factor *k* of 9.2 ± 0.5. Taking the shifts in voltage dependence into account, the kinetics of channel inactivation were comparable in both heterologous models but slightly slower in hSC-CM (**Figure 2E**).

**Figure 3:**
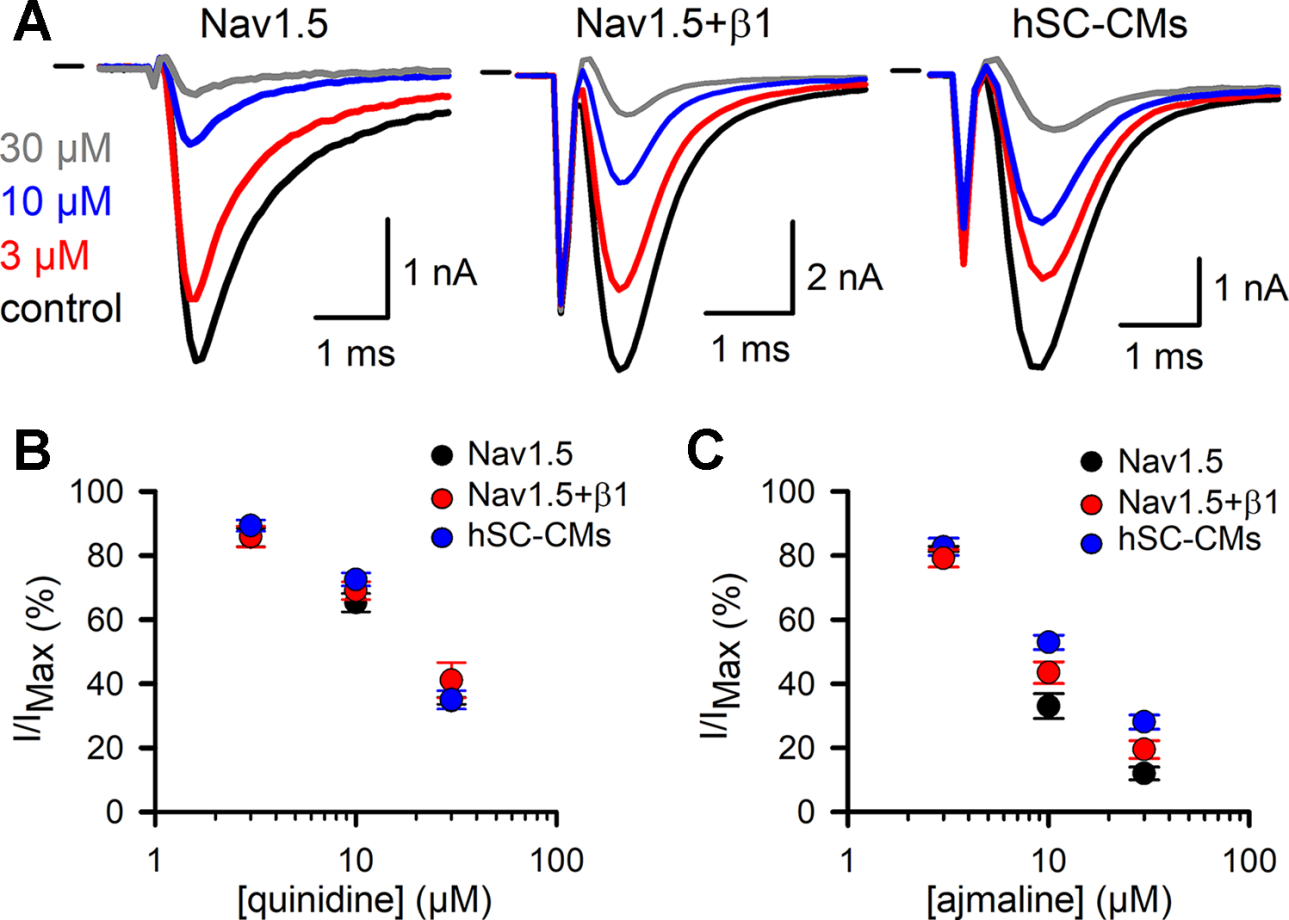
Quinidine and ajmaline I_Na_ drug response: **(A)** From left to right representative steady-state I_Na_ recordings of Nav1.5, Nav1.5+β1 and hSC-CM upon application of different ajmaline concentrations. Represented conditions are the current amplitudes in absence of drug (control condition, black trace), 3 (red), 10 (blue), and 30 µM (grey) ajmaline, respectively. Currents were elicited by repetitively pulsing to −10 mV as described in material and methods. Starting from the lowest to the highest concentration, every concentration was washed-in until a steady-state effect on the current was observed. Horizontal bar at the start represents the zero current level. **(B**, **C)** Concentration-effect curves obtained by plotting the normalized current (I/I_MAX_) as a function of applied quinidine (panel B) and ajmaline (panel C) concentration, respectively. For each drug the response of the three different models is shown; Nav1.5 (black circles, quinidine n = 8, ajmaline n = 6), Nav1.5+β1 (red circles, quinidine n = 7, ajmaline n = 7), and hSC-CMs (blue circles, quinidine n/#batch = 12/3, ajmaline n/#batch = 8/1).

The effect of the vehicle (0.1% DMSO) was evaluated and found not to modulate I_Na_ in either of the three models (**Supplemental Figure 2**), which agrees with what has been reported (Hyun et al., 2017). The development of drug effect was evaluated by repetitive application of a depolarizing voltage step (**Figure 3A**). For class Ia, the two drugs tested were quinidine and ajmaline. Normalizing the steady-state current after drug addition to the control current yielded concentration-inhibition curves. For quinidine, all three models displayed a similar response to the three concentrations tested and no differences (p-value > 0.01) were observed (**Figure 3B**, **Table 1**). For ajmaline, more inhibition was observed in Nav1.5 compared to both other models (**Figure 3C**). However, the reduced affinity observed upon coexpression of β1 was not sufficient to be statistically different from the Nav1.5 model. On the other hand, the response in the hSC-CMs model appeared to display a small but significant (p-value < 0.01) decreased sensitivity to the Nav1.5 model (**Table 1**). Since β1 coexpression reduced the affinity slightly, there was no statistical difference (p-value >0.01) in the amount of current inhibition between hSC-CMs and the Nav1.5+β1 model.

**Table 1:** Amount of current inhibition per drug concentration is represented for each model as mean % block ± S.E.M. % block per drug concentration was statistically evaluated to obtain p-values for differences in response between the models: Nav1.5+β1 vs. Nav1.5, hSC-CM vs. Nav1.5, and hSC-CM vs. Nav1.5+β1, respectively.

	Drug	µM				p-value		
			Nav1.5	Nav1.5+β1	hSC-CM	Nav1.5+β1	hSC-CM	hSC-CM
			% block	% block	% block	Nav1.5	Nav1.5	Nav1.5+β1
**Ia**	Quinidine	3	14 ± 3	14 ± 3	11 ± 2	not significant (0.266)		
		10	35 ± 3	31 ± 3	28 ± 2	not significant (0.114)		
		30	65 ± 1	59 ± 5	65 ± 3	not significant (0.388)		
	Ajmaline	3	18 ± 2	21 ± 3	17 ± 3	not significant (0.564)		
		10	67 ± 4	57 ± 3	47 ± 2	0.044	<0.001	0.055
		30	88 ± 2	81 ± 3	72 ± 2	0.029	<0.001	0.083
**Ib**	Lidocaine	3	20 ± 2	7 ± 1	7 ± 1	<0.001	<0.001	0.807
		10	31 ± 3	19 ± 2	12 ± 1	<0.001	<0.001	0.003
		30	56 ± 2	37 ± 2	26 ± 2	<0.001	<0.001	<0.001
		100	69 ± 1	56 ± 3	43 ± 3	0.009	<0.001	0.005
		300	87 ± 2	69 ± 3	52 ± 3	<0.001	<0.001	<0.001
		1000	89 ± 2	74 ± 3	72 ± 2	0.001	<0.001	0.531
	Phenytoin	3	11 ± 1	3 ± 1	4 ± 1	<0.001	<0.001	0.362
		10	17 ± 2	11 ± 2	6 ± 1	0.027	<0.001	0.027
		30	40 ± 2	21 ± 2	17 ± 1	<0.001	<0.001	0.150
		100	59 ± 2	36 ± 4	33 ± 2	<0.001	<0.001	0.449
		300	70 ± 2	48 ± 1	45 ± 3	<0.001	<0.001	0.071
	Phenythoin	3	27 ± 3	16 ± 5	11 ± 2	0.03	<0.001	0.195
	depolarised	10	50 ± 2	38 ± 5	39 ± 4	not significant (0.209)		
	Pre-pulse	100	76 ± 2	58 ± 5	58 ± 2	0.002	0.002	0.934
**Ic**	Flecainide	3	33 ± 3	28 ± 3	25 ± 2	not significant (0.058)		
		10	73 ± 2	61 ± 3	54 ± 2	0.001	<0.001	0.031
		30	89 ± 1	89 ± 1	83 ±2	not significant (0.012)		

Significance of difference is achieved if the value was <0.01. Not significant indicates that no significant difference between models was obtained with the p-value between parentheses.

**Figure 4:**
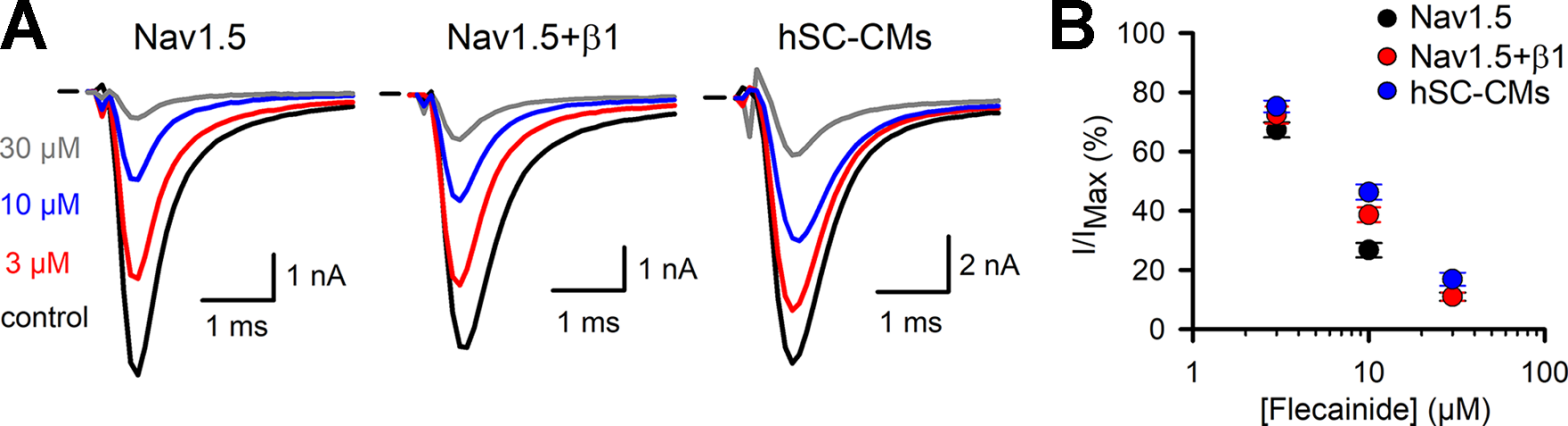
Flecainide I_Na_ drug response **(A)** From left to right representative steady-state I_Na_ recordings of Nav1.5, Nav1.5+β1 and hSC-CM elicited at −10 mV depolarization and presence of different flecainide concentrations. Represented conditions are the current amplitudes in absence of drug (control, black trace), 3 (red), 10 (blue), and 30 µM (grey) flecainide, respectively. Horizontal bar indicates zero current level. **(B)** Concentration-effect curves obtained by plotting the normalized current (I/I_MAX_) as a function of applied flecainide concentration. The drug response of the three different models are displayed, Nav1.5 (black, n = 16), Nav1.5+β1 (red, n = 14), and hSC-CMs (blue, n/#batch = 15/3).

Flecainide was selected as a representative drug of class Ic. For flecainide, the response appeared stronger in Nav1.5 compared to Nav1.5+β1 and hSC-CMs (**Figure 4B** and **Table 1**). However, the noticeable reduced response of cells expressing Nav1.5+β1 compared to cells expressing Nav1.5 was only statistically significant for the 10-µM concentration (**Figure 4D**). The lower sensitivity of hSC-CMs for flecainide was found to be statistically significant to the response in Nav1.5 (p-value <0.001) but not to Nav1.5+β1 (p-value >0.01). Thus, hSC-CMs displayed a flecainide sensitivity comparable to that of Nav1.5+β1.

**Figure 5:**
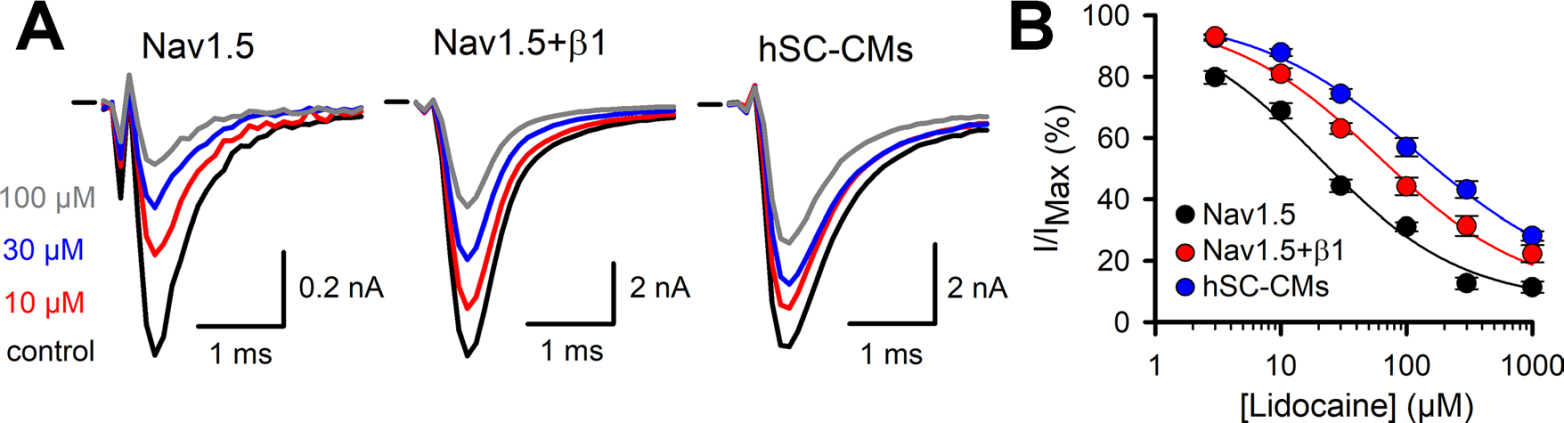
Lidocaine I_Na_ drug response **(A)** From left to right representative steady-state I_Na_ recordings of Nav1.5 (left), Nav1.5+β1 (middle), and hSC-CM (right) upon application of different lidocaine concentrations. Represented conditions are the current amplitudes in absence of drug (control, black trace), 10 (red), 30 (blue), and 100 µM (grey) lidocaine, respectively. Zero current level is indicated by the horizontal bar at the start. **(B)** The response (I/I_MAX_) to applied lidocaine concentration is shown for the three different models, Nav1.5 (black, n = 7), Nav1.5+β1 (red, n = 10), and hSC-CM (blue, n/#batch = 11/2). Note the difference in the response to lidocaine between Nav1.5 alone and in presence of β1.

While the previous drugs displayed no or rather small differences between the three models, the class Ib drugs lidocaine and phenytoin showed a more pronounced difference between Nav1.5 and the two other models. In our heterologous expression model, Nav1.5 alone displayed for lidocaine an estimated IC_50_ value of 20.4 ± 4.5 µM (n = 7, **Figure 5**), which is comparable to what has been reported (Hanck et al., 2000). Coexpression of β1 reduced the amount of current inhibition significantly at all concentrations tested (**Table 1**). Approximating the concentration-effect curve of Nav1.5+β1 yielded an estimated IC_50_ value of 59 ± 6.3 µM (n = 10). Besides the shift toward higher lidocaine concentrations, the maximal amount of inhibition was reduced from approximately 80% in Nav1.5 to 60% in Nav1.5+β1 (**Figure 5B**). Interestingly, the effect of lidocaine on I_Na_ of hSC-CMs was more similar to Nav1.5+β1 than to Nav1.5 alone. Likewise, the amount of current inhibition was at all concentrations significantly lower in hSC-CMs compared to Nav1.5 alone. In contrast, no significant difference in amount of I_Na_ inhibition was observed between hSC-CMs and Nav1.5+β1 (**Table 1**). hSC-CMs displayed an estimated IC_50_ of 116 ± 5 µM with a maximal inhibition of approximately 60% (n/#batch = 11/2). In neither of the models did lidocaine alter the kinetics of I_Na_ activation and inactivation (**Supplemental Table 1**).

**Figure 6:**
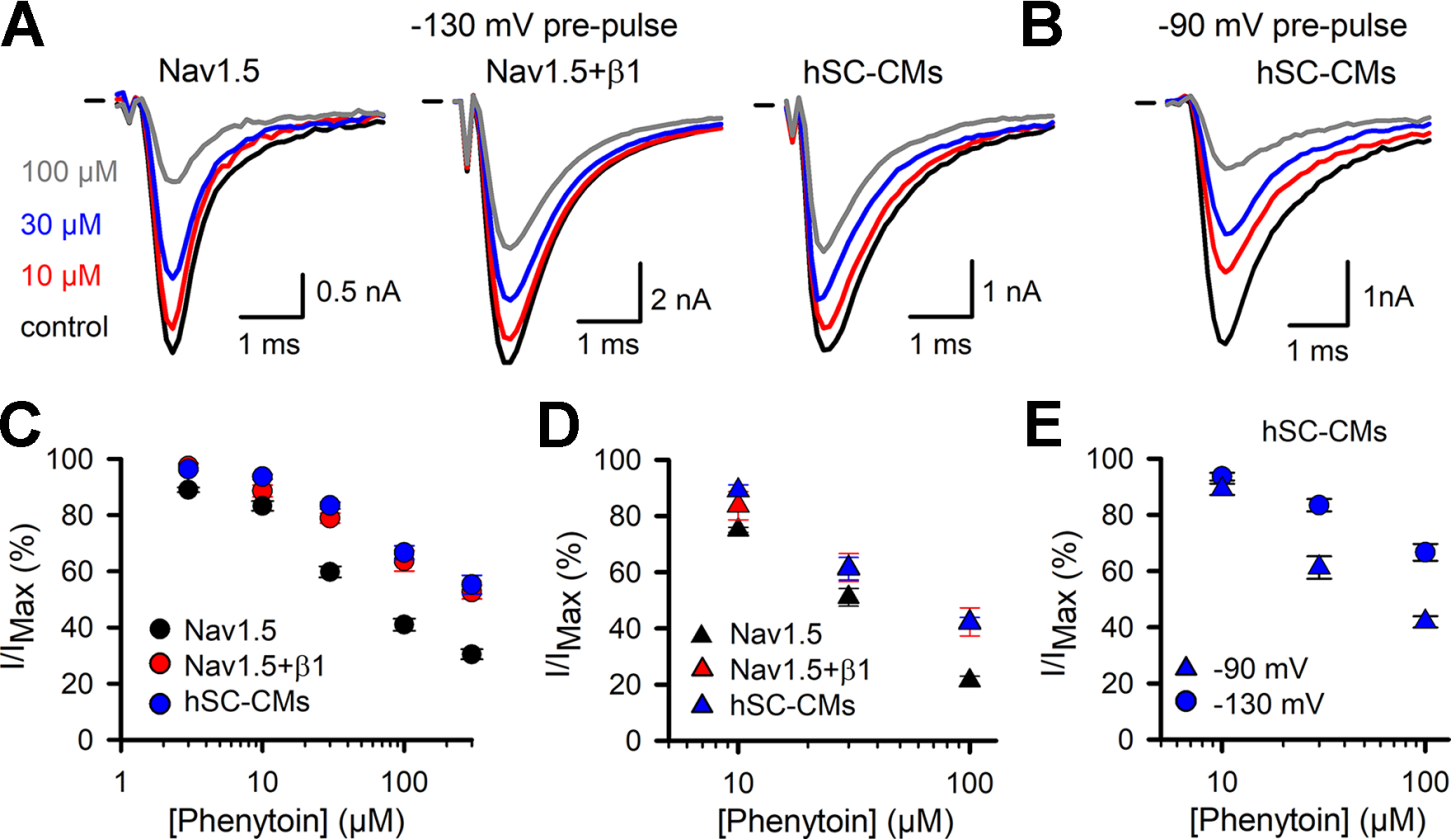
Phenytoin I_Na_ drug response **(A)** Representative current recordings from Nav1.5 (left), Nav1.5+β1 (middle), and hSC-CM (right) upon application of different phenytoin concentrations. Steady-state current amplitudes are shown for control condition (black), 10 (red), 30 (blue), and 100 µM phenytoin (grey), respectively. Zero current level is indicated by the horizontal bar at the start. **(B)** Representative current recordings from hSC-CM upon application of different phenytoin concentrations and with a more depolarized prepulse to −90 mV. Steady-state current amplitudes are shown for control condition (black), 10 (red), 30 (blue), and 100 µM phenytoin (grey), respectively. **(C)** The normalized current (I/I_MAX_) as a function of applied phenytoin concentration for Nav1.5 (black circle, n = 10), Nav1.5+β1 (red circle, n = 10), and hSC-CMs (blue circle, n/#batch = 12/3). **(D)** Concentration-effect curves obtained with a more depolarized prepulse (−100 mV for Nav1.5 and −90 mV for Nav1.5+β1 and hSC-CMs) are represented for Nav1.5 (black triangle, n = 5), Nav1.5+β1 (red triangle, n = 5), and hSC-CMs (blue triangle, n/#batch = 8/2). **(E)** Concentration-effect curves obtained for hSC-CMs at prepulse potentials of −130 (blue circle, retaken from panel **C**) or −90 mV (blue triangle, retaken from panel **D**).

Phenytoin was selected as second member of the Ib class of drugs. The response observed in the Nav1.5 model was in line with what has been reported, yielding an estimated IC_50_ of 30 ± 14 µM (Sanchez-Chapula and Josephson, 1983; Xu et al., 1991). Similar to what was observed for lidocaine, both Nav1.5+β1 and hSC-CM models displayed a significant reduced sensitivity to phenytoin compared to Nav1.5 alone (**Figure 6** and **Table 1**). At the highest concentration of 300 µM phenytoin tested, Nav1.5 displayed approximately 70% of current inhibition whereas in both Nav1.5+β1 and hSC-CM it did not yield more than 50%. Kinetics of activation and inactivation did not seem affected by phenytoin (**Supplemental Table 1**). However, it has been reported that I_Na_ inhibition by phenytoin depends on the potential of the conditioning prepulse due to a higher affinity of the drug for the inactivated state (Sanchez-Chapula and Josephson, 1983). Hereto, the development of current inhibition was re-evaluated using a more positive prepulse potential, e.g., −90 mV instead of −130 mV, to promote channel inactivation. As a potential of −90 mV was sufficient to induce channel inactivation, only a fraction (between 10% and 30%, **Figure 2D**) of the channels was available and the current activation elicited was already in control conditions less than using a prepulse of −130 mV. Nevertheless, the current amplitudes were sufficient to determine the amount of current inhibition for hSC-CM and Nav1.5+β1. Concentration-effect curves indicated that the amount of current inhibition was indeed increased with a prepulse potential of −90 mV compared to −130 mV. Since Nav1.5 alone displayed a more hyperpolarized voltage dependence of inactivation compared to both other models (**Figure 2D**), we decided to take this shift into account and used a −100-mV prepulse instead. Furthermore, with a prepulse of −90 mV, the noninactivated fraction of I_Na_ in Nav1.5 became insufficient in amplitude to determine channel inhibition accurately. Although the difference in I_Na_ inhibition of hSC-CMs and Nav1.5+β1 compared to Nav1.5 was smaller with more depolarized prepulse potentials (**Figure 6** and **Table 1**), the sensitivity of Nav1.5 remained a twofold higher and significantly different from both other models (p-value <0.01). The response of hSC-CM and Nav1.5+β1 was also with a more depolarized −90-mV prepulse similar and did not differ significantly from each other.

## Discussion

The aim of this study was i) to determine the contribution of β1 in the pharmacological responses to different classes of I_Na_ blockers and ii) to compare the affinity of known I_Na_ inhibitors between Nav1.5 +/− β1 expressed in heterologous cell lines and hSC-CMs (Cor.4U cardiomyocytes), cells now used extensively in high-throughput system safety derisking testing (Nozaki et al., 2014; Maddah et al., 2015; Zeng et al., 2016; Kopljar et al., 2018). In our study, using COS-7 cells, the I_Na_ density increased, as expected, in the presence of β1. The modulation of channel kinetics by β1 appeared to be minor but reported effects by others have been known to be variable (Isom et al., 1995; Makielski et al., 1996; Dhar Malhotra et al., 2001; Watanabe et al., 2008; Zhu et al., 2017). This variation may relate to differences in experimental setup such as the type of cells used (Moran et al., 2003). For the voltage dependence of activation and its kinetics we observed no marked differences between Nav1.5 and Nav1.5+β1, as has been reported (Makielski et al., 1996; Dhar Malhotra et al., 2001; Zhu et al., 2017). The voltage dependence of inactivation displayed, in the presence of β1, a small shift of approximately +6 mV toward more depolarized potentials without affecting the kinetics of inactivation. In literature, a similar shift in depolarization has been reported that varies between 5 (Dhar Malhotra et al., 2001) and 12 mV (Zhu et al., 2017). Recovery of inactivation was slightly accelerated with β1, as reported (Watanabe et al., 2008; Zhu et al., 2017).

The I_Na_ was well expressed in the hSC-CMs used, and the current amplitudes obtained were in line with what has been reported (Moreau et al., 2017). Voltage dependence of activation displayed a −10 mV shift to more hyperpolarized potentials compared to our heterologous Nav1.5 and Nav1.5+β1 models (**Figure 2D**) (Valdivia et al., 2002). On the other hand, the voltage dependence of inactivation was in hSC-CM shifted by 5 to 10 mV toward more depolarized potentials compared to Nav1.5+β1 or Nav1.5, respectively (**Figure 2D**). While the rate of inactivation did not show differences, the recovery process was slightly accelerated in hSC-CMs compared to Nav1.5 alone resembling the condition when both Nav1.5 and β1 are expressed. Thus, I_Na_ of hSC-CM resembled most to the I_Na_ generated in COS-7 cells expressing both Nav1.5+β1. Although the electrophysiological properties suggest that the I_Na_ in hSC-CMs is largely mediated by β1, it has to be taken into account that the β1 subunit is most likely not the only auxiliary subunit and other modulating processes can influence the kinetics of I_Na_ in hSC-CMs.

In order to use hSC-CM in safety pharmacology studies, it is important to know the effect of coexpressing β1 with Nav1.5 on the latter’s response to drugs. Quinidine (class Ia) inhibits Nav1.5 with a reported IC_50_ of 9.7 µM (Snyders et al., 1992; Harmer et al., 2011). Our results are in agreement and no difference in quinidine sensitivity was observed between the three models (**Figure 3** and **Table 1**). Ajmaline (class Ia) has a reported IC_50_ of approximately 27.8 µM, measured in rat ventricular cardiomyocytes (Bébarová et al., 2005). Using heterologously expressed I_Na_ in COS-7 cells, we observed slightly higher affinities as 10 µM inhibited more than 50% of the I_Na_ (**Table 1**). However, no significant differences were observed between Nav1.5 and Nav1.5+β1. hSC-CMs displayed a slightly reduced ajmaline sensitivity compared to Nav1.5 alone, with a difference of less than a twofold it comes close to the reported affinity in rat ventricular myocytes. Compared to Nav1.5+β1 the ajmaline response was in hSC-CMs similar.

Flecainide (class Ic) has a reported IC_50_ between 6 and 7 µM (Nitta et al., 1992; Ramos and Leary, 2004), which is comparable to our findings. Similar to the class Ia drugs, there was only a minor (not significant) difference in the response upon coexpression of β1 compared to Nav1.5 alone. Thus, likewise to the evaluated class Ia compounds, β1 did not affect flecainide affinity. When comparing the effect of flecainide in hSC-CMs to that of the heterologous models, hSC-CMs displayed a small but significant lower affinity compared to Nav1.5 at the 10-µM concentration. Compared to Nav1.5+β1 the response was similar in hSC-CMs (**Table 1**). Collectively, the data show that the responses of Nav1.5 to class Ia and Ic drugs is not markedly affected by β1 and the sensitivity of heterologous expression models and hSC-CMs is comparable.

On the other hand, the affinity of Nav1.5 for the class Ib drugs lidocaine and phenytoin was significantly decreased upon β1 expression. Lidocaine is a use-dependent inhibitor with a reported IC_50_ of 45 µM for the Nav1.5 channel (Bean et al., 1983; Bennett et al., 1995). We observed a similar response for Nav1.5 alone but the obtained inhibition was in both Nav1.5+β1 and hSC-CMs about a twofold to threefold lower (**Figure 5** and **Table 1**). A previous study using *Xenopus Laevis* oocytes as expression model, reported a similar finding and coexpression of β1 reduced Nav1.5’s lidocaine sensitivity (Makielski et al., 1996). To compare the lidocaine response of our three models to what is reported for native human myocytes, we need to consider that lidocaine is a use-dependent blocker and the pulse frequency will substantially determine the amount of I_Na_ block. Using a similar pulse protocol, evaluating I_Na_ inhibition at 1−2 Hz pulsing, 200 µM lidocaine gave between 20% and 35% of I_Na_ inhibition in human myocytes (Jia et al., 1993; Furukawa et al., 1995). In our conditions, heterologous expression of only Nav1.5 displays more than 80% inhibition whereas coexpression of β1 reduces it to approximately 65% (**Figure 5**). hSC-CMs displayed an even lower sensitivity, although not significant different from the Nav1.5+β1, and 200 µM lidocaine would inhibit around 55% of the I_Na_ (determined at 1 Hz pulsing, **Figure 5**). Thus, the lidocaine response of I_Na_ in hSC-CMs compares best to the reported effect in human myocytes but the affinity in hSC-CMs appears slightly higher.

Evaluating the inhibitory effect of phenytoin, a similar picture emerged and COS-7 cells coexpressing Nav1.5+β1 displayed a lower affinity than Nav1.5 alone. The hSC-CM model resembled the response of Nav1.5+β1 and both displayed a significant lower sensitivity compared to Nav1.5 (**Table 1**), which showed an affinity similar to what has been reported (Ragsdale et al., 1991). When the conditioning prepulse, used to recover I_Na_ inactivation, was more depolarized (i.e., from −130 to −90 mV for Nav1.5+β1 and hSC-CM or −100 mV for Nav1.5), the sensitivity to phenytoin and amount of I_Na_ inhibition increased. The increased sensitivity with a more depolarized voltage, or a more depolarized resting membrane potential, is explained by the observation that phenytoin is an inactivated state blocker, i.e., when channel inactivation is promoted, also channel inhibition is increased. Most likely, a similar finding can be predicted for lidocaine as this drug is also reported to bind the channel when it is inactivated (Lu et al., 2010).

Analyzing the pharmacological response of I_Na_, the hSC-CM model resembled best the heterologous Nav1.5+β1 model, supporting the fact that β1 forms part of the auxiliary subunits modulating the Nav1.5 channel in hSC-CM (Moreau et al., 2017). The β1 subunit modulates the inactivation kinetics of the Nav1.5 channel and, by doing so, it affects the sensitivity to drugs that bind the inactivated state, such as lidocaine and phenytoin. However, even when we compensated for the observed shift in voltage dependence of inactivation and compared the response with a prepulses to −100 mV for Nav1.5 and a prepulse to −90 mV for Nav1.5+β1 and hSC-CMs, a small significant difference remained between Nav1.5 and the other two models. This indicated that, besides the indirect effect of β1 on drug response by modulating the inactivation kinetics, β1 affects phenytoin sensitivity directly. A similar finding has been reported for lidocaine, whereby β1 reduces the sensitivity (Makielski et al., 1996).

Interestingly, Nav1.5’s affinity to mexiletine, a class Ib drug that shows structural similarity with lidocaine, has been shown to be affected by mutations that alter the activation of the voltage sensor of domain III (DIII-VSD) (Zhu et al., 2018). Structural data shows that the transmembrane helix of β1 is indeed in the vicinity of DIII-VSD (Yan et al., 2017; Pan et al., 2018), which hints toward a mechanism whereby β1 lowers lidocaine and phenytoin affinity by affecting DIII-VSD. Alternatively, because β1 binds with its extracellular part to the S5-S6 transmembrane segments of Domain I and IV (Makita et al., 1996), this interaction leads to conformational changes in the binding site(s) for both lidocaine and phenytoin that involves the S6 of Domain IV (Pless et al., 2011; Tikhonov and Zhorov, 2017; Nguyen et al., 2019).

In several cases, drug binding requires a specific channel conformation, e.g., the channel’s inactivated state. The occupation of certain states is dependent on the interpulse holding potential or in case of hSC-CM the resting membrane potential (RMP). As it is reported that the RMP of hSC-CMs is variable (Ma et al., 2011; Davis et al., 2012; Doss et al., 2012; Liang et al., 2016; Vaidyanathan et al., 2016; Chen et al., 2017; Hayano et al., 2017), this RMP variability needs to be considered when evaluating drug responses using techniques that do not control the RMP, which agrees with the observation that I_Na_ drug response is best determined under voltage-clamp condition (Goodrow et al., 2018). For open channel blockers, like some class Ia and Ic drugs, a more depolarized RMP means more channels are inactivated and the drug will not be able to block the channel. On the other hand, blockers with a high affinity for the inactivated state will exert a more pronounced effect. A recent study showed that phenytoin at 5 µM (i.e., a therapeutic concentration, used as anticonvulsant, not associated with cardiovascular effects in the clinic), sometimes affected the spontaneous beating of hSC-CMs causing in some experiments up to 50% beating arrest of the monolayer (Kopljar et al., 2018). These effects might originate from variability in RMP between different hSC-CM cultures, hereby affecting the amount of I_Na_ being blocked by phenytoin. In our setup, using voltage clamp, the interpulse holding potential was well controlled allowing for accurate assessment of the pharmacological response.

Overall, our data indicate that the pharmacological response for the five drugs evaluated on I_Na_ in hSC-CMs compares better to the response in heterologous expressed Nav1.5+β1 currents than currents composed of Nav1.5 alone. However, β1 is not the only interacting subunit and other auxiliary subunits of Nav1.5 (other β subunits and scaffolding proteins) are probably expressed in hSC-CM (Abriel, 2010). Furthermore, even other channels, such as the Kir2.1 K^+^ channel, can form complexes with Nav1.5 (Willis et al., 2015; Utrilla et al., 2017; Ponce-Balbuena et al., 2018). As these subunits and interactions can modulate the response of Nav1.5, the expression of them in hSC-CMs most likely explains the observed difference in I_Na_ kinetics between hSC-CMs and heterologous expressed Nav1.5+β1 (**Figure 1**). Furthermore, although Nav1.5 is the main α-subunit of the I_Na_ in hSC-CM, most likely other Nav channel subunits contribute to the total I_Na_ expressed (Moreau et al., 2017). For completeness, all these factors should be taken into account and the observed difference in drug response between hSC-CM and heterologous expressed Nav1.5 cannot be ascribed to β1 alone. Anyway, our data shows that i) the β1-subunit influences the affinity for class Ib drugs but not for the class Ia and Ic compounds evaluated and ii) the drug response of I_Na_ in hSC-CMs compares very well to I_Na_ of COS-7 cells expressing Nav1.5+β1.

## Data Availability Statement

The datasets generated for this study are available on request to the corresponding author.

## Author Contributions

Performed experiments: DVS. Set up project methodology: AT, DG, HL, IK, and AL. Data analysis: DVS, IK, and AL. Writing article: DVS, IK, AT, DG, DJS, HL, and AL.

## Conflict of Interest

Authors IK, AT, DG and HL were employed by Janssen Pharmaceutica, Beerse, Belgium.

The remaining authors declare that the research was conducted in the absence of any commercial or financial relationships that could be construed as a potential conflict of interest.
