# Supplementary material for: Pharmacological Profile of the Sodium Current in Human Stem Cell-Derived Cardiomyocytes Compares to Heterologous Nav1.5+β1 Model
Source: Front Pharmacol. 2019 Dec 11;10:1374. doi: 10.3389/fphar.2019.01374 (PMC6917651; doi:10.3389/fphar.2019.01374)
Supplement: Supplementary file 1 [file DataSheet_1.pdf]

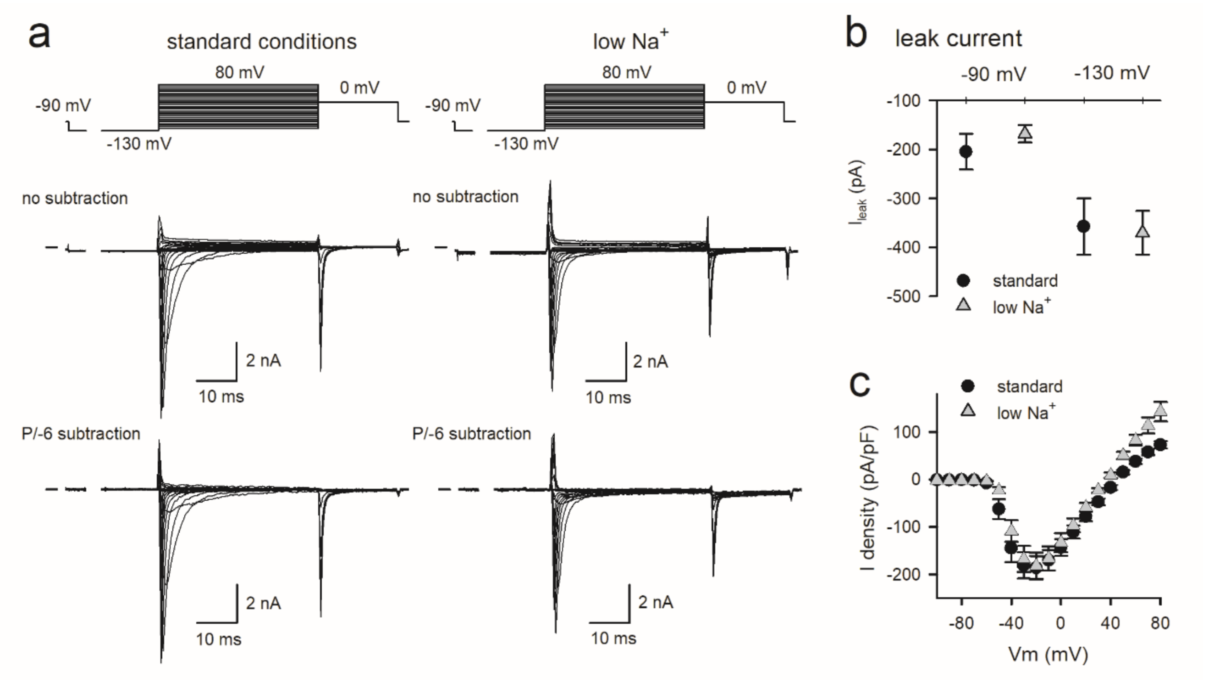

Supplemental Figure 1. Comparison of I_{Na} recordings in hSC-CMS obtained using ECS and ICS solutions with standard or lowered ion concentrations. **a**) Panel shows representative I_{Na} recordings obtained in standard conditions (currents on the left) or in solutions with lowered ion concentrations (currents on the right, indicated by low Na^+). Composition of the standard ECS and ICS solutions is described in the methods section. The solutions adapted for lowering Na^+ concentration were composed of; ECS (in mM): 30 NaCl, 5 KCl, 1.8 $CaCl_2$, 1 $MgCl_2$, 15 glucose, 15 HEPES, 1 Na pyruvate, 120 NMDG, pH 7.4 using HCl; ICS (in mM): 30 KCl, 10 HEPES, 5 $MgCl_2$, 5 NaCl, 2 $CaCl_2$, 5 EGTA, 120 CsCl, pH 7.2 using KOH. The top I_{Na} recordings were elicited with the pulse protocol shown on top and are non-subtracted tracings. The I_{Na} recordings shown at the bottom are the traces after a P/-6 subtraction, which was used to subtract capacitive currents. Horizontal bar at the start of the I_{Na} recordings indicates the zero current level. **b**) Plot shows the average background leak current amplitudes ± S.E.M. at -90 mV and -130 mV in standard conditions (black circles, n = 24) versus low Na^+ solutions (grey triangles, n = 11). Leak current amplitudes were obtained from non-subtracted I_{Na} recordings as shown in panel a. Note that the background leak was in both recording conditions similar and the amplitudes were in the order that they did not impose voltage-clamp problems. **c)** Current density versus voltage curves for I_{Na} recorded in standard solutions (black circles, n = 15) or in low Na^+ solutions (grey triangles, n = 7). The peak amplitude of the I_{Na} activations from P/-6 subtracted tracings, as shown in panel a, were normalized to the cell capacitance and plotted as a function of depolarizing potential.

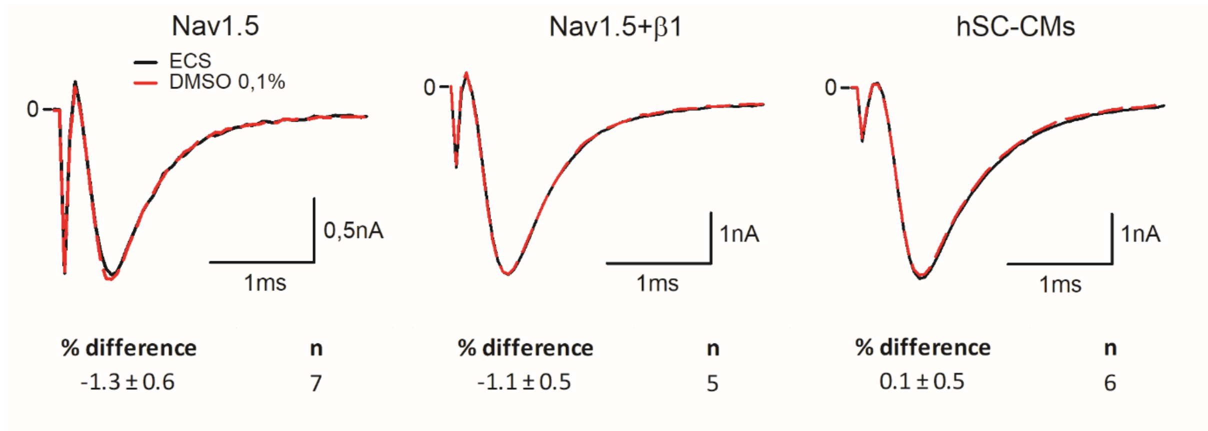

Supplemental Figure 2. Evaluation of the vehicle DMSO 0.1% on I_{Na} in the three models tested (COS-7 cells expressing either Nav1.5 or Nav1.5+β1 and hSC-CMs). Representative I_{Na} recordings obtained with standard solutions before (black trace) and after approximately 2 minutes exposure to DMSO 0.1% (red). Currents were elicited with a 40ms test pulse to -10 mV and shown traces are P/-6 subtracted. DMSO 0.1% was applied using the fast perfusion system. Percentage change in peak I_{Na} amplitudes by DMSO 0.1% was calculated by normalizing the I_{Na} upon 2 minutes wash-in of DMSO 0.1% to the I_{Na} in control conditions (before the start of DMSO 0.1% application). Percentage changes are represented as average ± S.E.M. below the representative I_{Na} recording (n indicates the number of cells analysed).

	Nav1.5		Nav1.5+β1		hSC-CM	
	Tau (ms)	n	Tau (ms)	n	Tau (ms)	n/batch
control ACT	0.33 ± 0.05	4	0.22 ± 0.04	7	0.23 ± 0.04	6/1
Lidocaine ACT	0.38 ± 0.08		0.20 ± 0.03		0.29 ± 0.06	
control IN	0.41 ± 0.02		0.41 ± 0.04		0.43 ± 0.04	
Lidocaine IN	0.38 ± 0.02		0.36 ± 0.05		0.37 ± 0.05	
control ACT	0.26 ± 0.06	6	0.28 ± 0.05	6	0.18 ± 0.04	6/1
Phenytoin ACT	0.27 ± 0.04		0.28 ± 0.05		0.21 ± 0.04	
control IN	0.43 ± 0.05		0.39 ± 0.02		0.39 ± 0.05	
Phenytoin IN	0.40 ± 0.03		0.40 ± 0.02		0.40 ± 0.05	

Supplemental table 1. Activation and inactivation kinetics of I_{Na} at -10 mV in presence of lidocaine or phenytoin. Represented activation (ACT) and inactivation (IN) time constants were obtained by approximating either the activating or the inactivating part of I_{Na} in control condition and upon steady-state wash-in of lidocaine or phenytoin, from recordings as shown in figure 5a and 6a, with a single exponential function. Results are represented as mean ± S.E.M. with n the number of cells analysed. For hSC-CM both the number of cells and batches is represented. There were no significant differences in the kinetics between those of control conditions and upon steady-state wash-in of with lidocaine (1000 µM) or phenytoin (300 µM).
